# Vitamin D Deficiency in Kazakhstani Children: Insights from a Systematic Review and Meta-Analysis

**DOI:** 10.3390/medicina61030428

**Published:** 2025-02-28

**Authors:** Indira Karibayeva, Galiya Bilibayeva, Assiya Iglikova, Aya Yerzhanova, Roza Alekesheva, Makhigul Maxudova, Neilya Ussebayeva

**Affiliations:** 1 Department of Health Policy and Community Health, Jiann-Ping Hsu College of Public Health, Georgia Southern University, Statesboro, GA 30460, USA; 2 Department of Nursing, Faculty of Medicine and Public Health, Al-Farabi Kazakh National University, Almaty 050038, Kazakhstan; bilibayeva@kaznu.kz (G.B.); iglikova-a@mail.ru (A.I.); yerzhanova.aya@kaznu.kz (A.Y.); alekesheva@kaznu.kz (R.A.); maksudova63@gmail.com (M.M.); usebaeva@mail.ru (N.U.)

**Keywords:** vitamin D, vitamin D deficiency, children, infants, prevalence, Kazakhstan, national program, systematic review, meta-analysis

## Abstract

*Background and Objectives*: Kazakhstan’s unique geographic, dietary, and cultural factors contribute to the widespread occurrence of vitamin D deficiency across the entire country population, particularly among children. This study aims to assess the mean prevalence of vitamin D deficiency in children in Kazakhstan and determine whether it differs between healthy and non-healthy children, as well as between infants and older age groups. *Materials and Methods*: A comprehensive literature search was performed across five databases by two researchers. Studies were eligible if they were observational and provided the number of children with serum 25-hydroxyvitamin D levels below 20 ng/mL out of the total number of children assessed in Kazakhstan. *Results*: Eleven studies were included in the analysis, assessing 1396 children, of whom 714 had the outcome of interest. The pooled mean estimate of vitamin D deficiency among children was 56% (95% CI, 46–65%), with particularly concerning rates among infants at 65% (95% CI, 44–82%). No substantial differences were observed between healthy and non-healthy children. *Conclusions*: The prevalence of vitamin D deficiency among children is alarmingly high. These results highlight the urgent necessity of tackling vitamin D deficiency as a public health priority. Incorporating vitamin D deficiency prevention into Kazakhstan’s national healthcare program is vital for improving child health outcomes and reducing the long-term burden of associated complications.

## 1. Introduction

Vitamin D plays a critical role in the growth and development of children, influencing skeletal health, immune function, and overall well-being [1]. Sufficient vitamin D levels are crucial for the absorption of calcium and the process of bone mineralization [2,3]. The most common impacts of vitamin D deficiency (VDD) involve bone and musculoskeletal health, leading to rickets in children and osteomalacia in adults. Beyond bone health, VDD has been associated with several health complications in pregnant women, including pre-eclampsia, pregnancy-induced diabetes, premature birth, and postpartum mood disorder [4]. In infants and children, it is linked to low birth weight, reduced bone density, respiratory tract infections, and worsening asthma symptoms [5,6]. VDD in infants and children is also linked to increased autoimmunity and allergies [7]. Given the vulnerability of children to VDD due to their physiological growth demands, understanding its prevalence is vital for developing effective prevention and management strategies.

Kazakhstan’s unique geographical, dietary, and cultural characteristics may contribute significantly to VDD among its population, particularly children [8,9,10]. The country’s vast territory experiences long winters and limited sunlight exposure during the colder months, which restricts the synthesis of vitamin D through the skin. Additionally, traditional dietary patterns in Kazakhstan often lack sufficient vitamin D intake sources, such as high-fat fish, and dairy products with added nutrients [11,12]. Cultural norms, including modest clothing styles in some communities, may further limit sun exposure, exacerbating the risk of deficiency [13]. A systematic review of VDD among adults in Kazakhstan reported alarmingly high rates of 57% [14]. These combined factors may contribute to the high prevalence of VDD, especially among vulnerable groups like infants and children.

Despite these known risk factors, there is a notable lack of comprehensive data on VDD among children in Kazakhstan. While several studies have examined vitamin D levels in the Kazakhstani population, data specific to children remain scarce and not systemic. Furthermore, no specific guidelines exist for the prevention and treatment of VDD in children in Kazakhstan, aside from those addressing rickets [15]. This gap in research and evidence is concerning, given the critical developmental window during childhood and the potential long-term health consequences of deficiency. Previous studies conducted in other countries have demonstrated the prevalence and determinants of VDD, but similar data specific to Kazakhstan’s unique context is insufficiently explored [16,17,18]. This lack of evidence hinders the formulation of targeted public health interventions and policies.

Addressing this research gap is imperative to guide public health initiatives in Kazakhstan. The aim of this study is to evaluate the mean prevalence of VDD among children in Kazakhstan and examine whether it differs between healthy and non-healthy children, as well as between infants and older children.

## 2. Materials and Methods

### 2.1. Search Strategy Framework

An initial search of the PROSPERO international database for registered studies on similar topics identified a review protocol focusing on the mean vitamin D levels in Kazakhstan and another protocol examining VDD levels specifically among adults in Kazakhstan [19,20]. To assess the mean prevalence of VDD among children in Kazakhstan, this systematic review protocol was registered in the PROSPERO international database (PROSPERO ID: CRD42025635124). Subsequently, a comprehensive search was conducted across five databases: PubMed, ScienceDirect, Scopus, Web of Science, and Google Scholar. The search strategy employed the keywords “vitamin D” AND “Kazakhstan”. No restrictions were applied regarding publication year, but the search was limited to studies published in English and Russian, focusing on humans. The search was performed between 1 November 2024 and 31 December 2024. Additional filters were applied, including the selection of research articles and the exclusion of other types of publications. In Google Scholar, the search was restricted to titles only.

### 2.2. Inclusion and Exclusion Criteria of Studies and Data Extraction

To determine the eligibility of the retrieved articles, pre-specified inclusion and exclusion criteria were applied, guided by the PICOS framework [21]. Population (P): Studies focusing on children aged 0 to 18 years in Kazakhstan. Intervention/Exposure (I): Serum 25-hydroxyvitamin D (25(OH)D) levels were measured to determine vitamin D status, with vitamin D deficiency (VDD) defined as a serum 25(OH)D level below 20 ng/mL. Comparison (C): Studies focusing on healthy children or children with acute or chronic conditions. A secondary comparison was made based on different age groups. Outcomes (O): Studies reporting the prevalence or number of children with VDD, along with data on the total number of children assessed. Study Design (S): Observational studies, including cohort studies with cross-sectional data, cross-sectional studies, and case–control studies published in English or Russian. The exclusion criteria included studies that focused exclusively on children with VDD or those that included a matched number of vitamin D-deficient and non-deficient children; studies reporting only the mean serum 25(OH)D levels without additional relevant data; studies with duplicate results already included in the analysis; studies examining the adult population in Kazakhstan; reviews, conference proceedings, abstracts, case reports, editorials, and commentaries; and studies using different cut-off values to define VDD.

The review and synthesis of the literature were carried out in accordance with the Preferred Reporting Items for Systematic Reviews and Meta-Analyses (PRISMA) guidelines [22]. After removing duplicates, two authors autonomously assessed the eligibility of the studies based on the title and abstract of the search results. Full-text articles that satisfied the preliminary screening criteria were then evaluated against the established eligibility criteria for data extraction. Data extracted from the chosen studies included the last name of the first author, publication year, study city or district, characteristics of the study population (e.g., specific condition or healthy controls), sample size, number of boys, age in years or in months, 25(OH)D levels in blood if provided, 25(OH)D level assessment method, and the number of children diagnosed with VDD. The data extraction process was conducted autonomously by two researchers responsible for data extraction, and any disagreements were clarified through consultation with a third author to achieve consensus. This collaborative approach ensured accuracy and consistency in study selection and data extraction.

### 2.3. Risk of Bias Assessment

The risk of bias assessment was carried out using the Newcastle–Ottawa Scale (NOS), which is specifically designed for case–control studies, as well as its revised form for cross-sectional studies [23]. This scale evaluated each study based on eight criteria across three domains: selection, assessed with four criteria; comparability, assessed with one criterion; and exposure, assessed with three criteria. The adapted version for cross-sectional studies evaluated each study based on six criteria divided into three main domains: selection, assessed with three criteria; comparability, assessed with one criterion; and exposure, assessed with two criteria. Each criterion was given a score of up to one point, with the comparability criterion capable of earning two points. The overall score ranged from 0 to 9 for case–control studies and from 0 to 7 for cross-sectional studies, with higher scores indicating better study quality. Two authors (IK and GB) independently assessed the quality of the included studies, and a third author (NU) calculated the inter-rater agreement between them. Case–control studies that achieved a score of seven or higher, and cross-sectional studies with five points or more, were included in the review.

### 2.4. Statistical Strategy for Data Synthesis

The RStudio software (version 2024.12.0), along with the ‘metafor’ and ‘meta’ packages, was utilized to determine the pooled mean prevalence of VDD among children in Kazakhstan. This was performed employing a random-effects model for prevalence meta-analysis, with 95% confidence intervals [24,25]. Forest plots were produced to visually represent the pooled analysis results. A meta-regression analysis based on the year of publication and the number of boys in the study was applied to assess heterogeneity. To further explore the sources of heterogeneity, an influence analysis and leave-one-out analysis were performed [25]. The study’s generalizability was examined through a publication bias assessment, which involved visually inspecting a drapery plot and conducting statistical analysis with Egger’s test. A subgroup analysis was used to compare healthy children with those who had health conditions and to analyze differences between infants and older children [25].

## 3. Results

### 3.1. Included Study Characteristics

**Figure 1:**
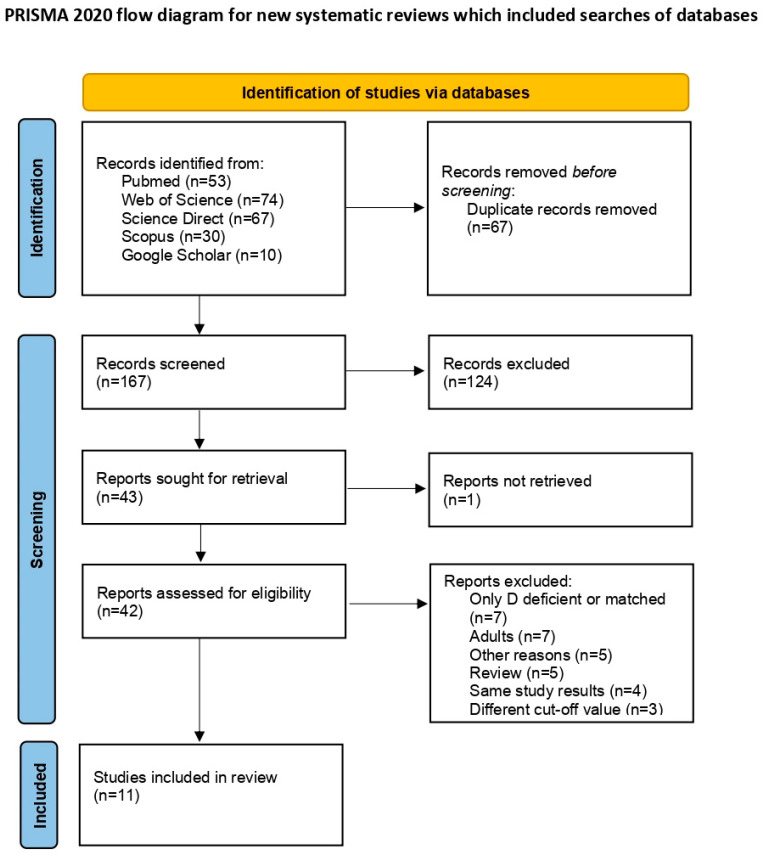
PRISMA Flow diagram of Study Selection.

The applied search strategy resulted in 234 articles. After removing 67 duplicates, 167 non-duplicative search results were initially screened, and 124 titles were deemed ineligible for full-text review. Out of 43 remaining articles, 42 articles underwent full-text review, as one article’s full text was not available. Upon full assessment, eleven studies met the inclusion criteria. Seven studies focused on adults, five studies were reviews, and five were excluded for other reasons. Seven studies were excluded because they either included only children with VDD or included a matched number of vitamin D-deficient children and non-deficient children [26,27,28,29,30,31,32]. Additionally, four studies presented the same results as studies already included in the review [33,34,35,36]. In three of the excluded studies, VDD was defined as serum 25(OH)D levels below 30 ng/mL or below 25 ng/mL [37,38,39]. Figure 1 displays the PRISMA flow diagram [22].

The selected eleven studies were published between 2020 and 2024. Three studies were conducted in Aktobe, Karaganda, and Astana, respectively, while one study was conducted in Almaty and Semey. The methodological designs included five case–control studies, five cross-sectional studies, and one cohort study. Among these, three studies focused on patients with chronic kidney disease, while one study examined cancer patients, recurrent respiratory infection (RRI) patients, congenital pneumonia cases, diabetic nephropathy, and patients infected with *Helicobacter pylori* (*H. pylori*). Additionally, five studies included healthy patient groups. Three of the included studies focused exclusively on infants, while the remaining studies examined children across various age groups. In total, 1396 children were assessed across the eleven studies. Of these, 714 children were found to have serum 25(OH)D levels below 20 ng/mL, classifying them as vitamin D deficient. In seven out of eleven studies, the method for serum 25(OH)D level assessment was not provided. Further details regarding the selected and analyzed articles are presented in Table 1.

### 3.2. Meta-Analysis of Vitamin D Deficiency Prevalence

**Figure 2:**
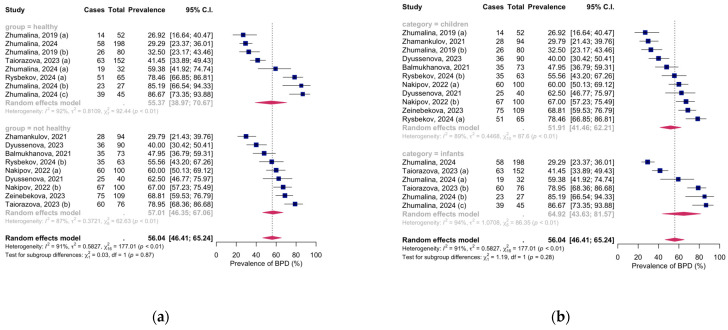
Vitamin D Deficiency Rates Among Kazakhstani Children: (**a**) based on health conditions; (**b**) based on age groups. Abbreviations: CI—confidence interval; Zhumalina, 2019 (a) [40]—environmentally friendly region; Zhumalina, 2019 (b) [40]—oil and gas producing region; Nakipov, 2022 (a) [44]—children with oncology, no nutritional support; Nakipov, 2022 (b) [44]—children with oncology, with nutritional support; Taiorazova, 2023 (a) [46]—healthy; Taiorazova, 2023 (b) [46]—diabetic nephropathy; Rysbekov, 2024 (a) [48]—children with no *H. Pylori*; Rysbekov, 2024 (b) [48]—children with *H. Pylori*; Zhumalina, 2024 (a) [50]—7–12 months; Zhumalina, 2024 (b) [50]—1–6 months; Zhumalina, 2024 (c) [50]—0–28 days.

Eleven studies, with seventeen groups, presented data on the prevalence of VDD among children in Kazakhstan. Analysis of five studies with eight groups shows that the pooled mean estimate of VDD in healthy children was 55% (95% CI, 39–71%), with high heterogeneity: I^2^ = 92%, Q (df = 7) = 92, *p* < 0.01. Analysis of eight studies with nine groups shows that the pooled mean estimate of VDD in children with health conditions was 57% (95% CI, 46–67%), with high heterogeneity: I^2^ = 87%, Q (df = 8) = 63, *p* < 0.01 (Figure 2a).

Additionally, an analysis of eight studies with eleven groups shows that the pooled mean estimate of VDD in children of various ages was 52% (95% CI, 41–62%), with high heterogeneity: I^2^ = 89%, Q (df = 10) = 88, *p* < 0.01. Analysis of three studies with six groups shows that the pooled mean estimate of VDD in infants was 65% (95% CI, 44–82%), with high heterogeneity: I^2^ = 94%, Q (df = 5) = 86, *p* < 0.01. The total pooled mean estimate of VDD in children was 56% (95% CI, 46–65%), I^2^ = 91%, Q (df = 16) = 177, *p* < 0.01 (Figure 2b).

**Figure 3:**
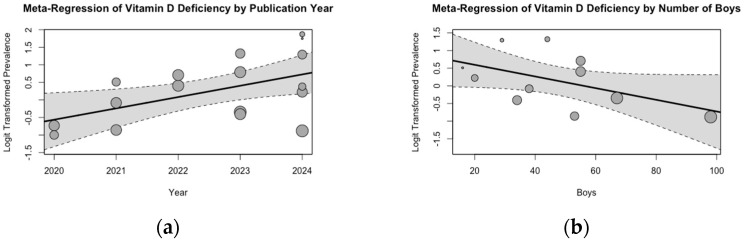
Meta-Regression Analysis of the Pooled Mean VDD Estimate in Children: (**a**) by Year of Publication; (**b**) by Number of Boys.

A meta-regression analysis demonstrated a significant positive association between publication year and the pooled mean estimate of VDD. Specifically, more recent studies reported a higher prevalence of VDD, a finding that was statistically significant (*p* < 0.01) (Figure 3a). A second meta-regression analysis explored the influence of gender on the pooled estimate of VDD. This analysis suggested that an increase in the proportion of male participants was associated with a decrease in the pooled mean prevalence of VDD. However, this relationship was not statistically significant (*p* = 0.07) (Figure 3b).

**Figure 4:**
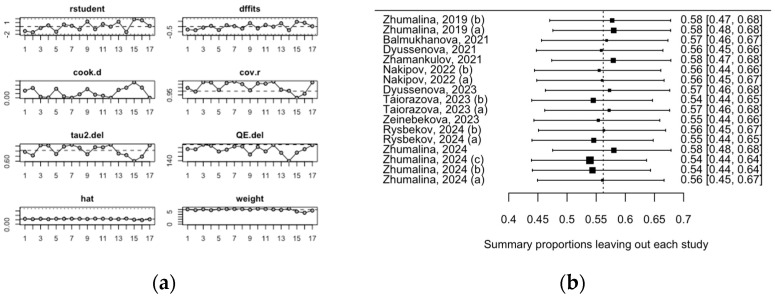
Heterogeneity Assessment of the Pooled Mean VDD Estimate in Children: (**a**) Influence Analysis; (**b**) Leave-One-Out Analysis.

An influence analysis and leave-one-out analysis did not identify any studies that significantly impacted the pooled estimate of the prevalence of VDD among children in Kazakhstan (Figure 4a,b).

**Figure 5:**
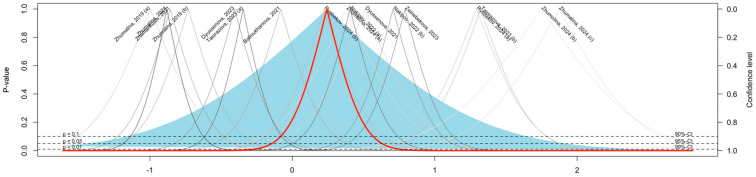
Drapery Plot of the Publication Bias Assessment.

Figure 5 presents the drapery plot of the publication bias assessment with no evident asymmetry. The lack of publication bias was additionally validated through the non-significant findings of Egger’s test for publication bias (*p* > 0.05).

### 3.3. Evaluation of Risk of Bias

The results of the risk of bias evaluation are outlined in Table 2, separately for case–control and cross-sectional studies, according to the NOS. All the included case–control studies achieved a NOS score of 7 or higher out of a possible 8, while all cross-sectional studies obtained a minimum score of 5 out of 7. These scores indicate that the included studies were of high quality with a low risk of bias. Consequently, they were included in the present systematic review and meta-analysis.

## 4. Discussion

### 4.1. Main Findings of the Present Study and Their Practical Implications

This systematic review and meta-analysis sought to evaluate the prevalence of VDD, defined as a serum 25(OH)D level below 20 ng/mL, among children in Kazakhstan. The findings reveal an alarmingly high prevalence, with the total pooled mean estimate of VDD in children in Kazakhstan at 56% (95% CI, 46–65%). Notably, the prevalence did not significantly differ based on health status; VDD rates were nearly identical among healthy and non-healthy children, at 55% (95% CI, 39–71%) and 57% (95% CI, 46–67%), respectively. However, age emerged as a critical determinant of VDD. Infants demonstrated a substantially higher prevalence, with almost two-thirds being vitamin D deficient at 65% (95% CI, 44–82%) compared to children of various older age groups, where the prevalence was 52% (95% CI, 41–62%).

These findings are consistent with trends observed in the global literature. For instance, a recent meta-analysis on VDD among healthy children in Iran, using the same deficiency threshold, reported a prevalence of 31% (95% CI, 30–31%), markedly lower than that observed in Kazakhstan [16]. By contrast, a meta-analysis examining VDD among neonates in Turkey revealed even higher rates of 87% (95% CI, 70–95%) [18]. Similarly, a meta-analysis conducted in Africa, utilizing a higher cutoff for deficiency (serum 25(OH)D level below 30 ng/mL), reported a prevalence of 64% (95% CI, 9–100%) among newborns [17]. Importantly, these studies underscore the significant correlation between maternal and neonatal vitamin D levels, a finding corroborated by our prior meta-analysis on adults in Kazakhstan, which reported a similarly high prevalence of VDD (57%; 95% CI, 45–69%) [14]. These consistent patterns highlight the widespread nature of VDD across different demographic groups and emphasize the need for targeted interventions.

The high prevalence of VDD, particularly among infants, underscores the urgent need for targeted interventions to address this public health challenge in Kazakhstan. While the national healthcare development program outlines a comprehensive strategy to prevent and manage non-communicable diseases by addressing key risk factors such as hypertension, obesity, diabetes, and unhealthy lifestyle behaviors, the omission of VDD prevention is a significant gap [51]. Given the critical role of vitamin D in bone health, immune function, and overall well-being, particularly for children and pregnant women, this omission warrants immediate attention [2,3]. Moreover, recent evidence links VDD to an increased risk of respiratory tract infections, impaired growth, and poor dental health in children, emphasizing the broader health implications of the deficiency [52,53,54]. Furthermore, emerging evidence highlights the broader implications of maternal VDD, including its impact on neurocognitive development in offspring. For example, Melough et al. (2020) demonstrated that maternal plasma 25(OH)D levels during gestation are positively associated with neurocognitive development in children, underscoring the importance of addressing VDD in pregnant women to mitigate long-term developmental risks [55].

To address this gap, Kazakhstan should integrate strategies for the prevention and management of VDD into its national healthcare development program. Successful examples from other countries provide actionable models. Finland, for instance, implemented mandatory food fortification policies that significantly reduced population-level VDD [56]. In Poland, updated guidelines on the prevention and treatment of VDD emphasize daily supplementation of cholecalciferol for high-risk populations and provide recommendations for sun exposure [57]. In the United States, the Office of Dietary Supplements successfully led the National Vitamin D Initiative from 2004 to 2018, promoting awareness and prevention efforts [58,59]. Additionally, a national supplementation program for vitamin D in Iran demonstrated efficacy in reducing the prevalence of VDD and highlighted the importance of a prevention-based approach over a treatment-focused one [60,61,62]. Similarly, Canada has established robust supplementation programs targeting high-risk populations, such as infants, pregnant women, and older adults, achieving measurable reductions in deficiency rates and associated health complications [63,64].

Screening programs for pregnant women should also be prioritized in Kazakhstan, as maternal vitamin D status directly impacts neonatal and infant outcomes [4,5]. Public health campaigns promoting awareness of vitamin D’s importance, along with educational initiatives about adequate sunlight exposure and dietary intake, are essential. Collaborative efforts between healthcare providers, policymakers, and researchers are necessary to design evidence-based guidelines that address VDD comprehensively. Such initiatives will not only improve child health outcomes but also mitigate the long-term burden of health complications associated with this preventable condition.

### 4.2. Limitations

First, we were unable to analyze region-specific data or stratify the findings based on participants’ specific health conditions due to the limited number of studies included in this analysis. Consequently, the heterogeneity in selected article populations and settings may have influenced the pooled estimates. Second, limitations inherent in our previous meta-analysis on adults, such as the absence of analyses accounting for metabolic conditions, the seasonality of vitamin D measurements, and the amount of sun exposure among participants, also apply to the present meta-analysis [14]. Finally, most studies did not specify the assay method used to measure serum 25(OH)D. Among those that did, there was considerable variability in the techniques employed. Differences in reagents, assay sensitivity, and specificity are essential in the interpretation of the accuracy and comparability of vitamin D measurements [65,66].

To mitigate potential biases and ensure methodological rigor, we adhered to a standardized protocol throughout the study. We used pre-defined eligibility criteria and a structured data extraction table to minimize subjectivity during study selection, data extraction, and interpretation. Furthermore, we followed the PRISMA guidelines for conducting systematic reviews at every step of the process and transparently reported the methods used [22]. These measures strengthen the reliability of our findings, despite the inherent limitations of the available data.

### 4.3. Future Research Directions

To address the limitations of this study and build a more comprehensive understanding of VDD in Kazakhstan, future research should focus on:

- Investigating the role of metabolic disorders, seasonality, and sun exposure on vitamin D levels in children of varying ages.
- Assessing the prevalence of maternal VDD in Kazakhstan.
- Evaluating the efficacy of maternal interventions, such as screening and supplementation, on reducing VDD in newborns.
- Assessing the sensitivity and specificity of assays used

## 5. Conclusions

This meta-analysis highlights an alarmingly high prevalence of VDD among children in Kazakhstan at 56% (95% CI, 46–65%), with particularly concerning rates among infants at 65% (95% CI, 44–82%). These findings align with global evidence and underscore the critical need to address VDD as a public health priority. Incorporating VDD prevention into Kazakhstan’s national healthcare program is vital to improving child health outcomes and reducing the long-term burden of associated complications. A comprehensive upstream approach that combines supplementation with broader public health initiatives will be key to reducing VDD prevalence and protecting future generations.

## Figures and Tables

**Table 1:** Overview of the Included Studies.

First Author, Year	Region	Study Design	Groups	Total	Boys	Age (Mean ± SD) or Range	25(OH)D (ng/mL) (Mean ± SD)	25(OH)D Assessment	VDD (%)
Zhumalina, 2019 [40]	Aktobe	Case–control	Healthy, Kobda (a) Healthy, Kenkiyak (b)	52 (a) 80 (b)	n/a n/a	8–17 years (a) 8–17 years (b)	28 ± 11 (a) 24 ± 12 (b)	n/a	14 (27) (a) 26 (33) (b)
Balmukhanova, 2021 [41]	Almaty	Cross-sectional	CKD	73	38	2–18 years	Varied-based CKD stage	n/a	35 (48)
Dyussenova, 2021 [42]	Karaganda	Cross-sectional	CKD	40	16	1–17 years	n/a	ELISA	25 (62)
Zhamankulov, 2021 [43]	Astana	Cross-sectional	RRI	94	53	5.8 ± 3.3 years	31 ± 3	n/a	28 (30)
Nakipov, 2022 [44]	Astana	Cross-sectional data from cohort	Cancer, control (a) Cancer, nutritional intervention (b)	100 (a) 100 (b)	55 (a) 55 (b)	0–17 years	27 ± 12	n/a	60 (60) (a) 67 (67) (b)
Dyussenova, 2023 [45]	Karaganda	Case–control	CKD	90	34	1–17 years	n/a	n/a	36 (40)
Taiorazova, 2023 [46]	Semey	Case–control	Healthy (a) Congenital pneumonia (b)	152 (a) 76 (b)	67 (a) 44 (b)	newborn	21 ± 6 (a) 12 ± 7 (b)	Demeditec 25-OH Vitamin D total ELISA	63 (41) (a) 60 (79) (b)
Zeinebekova, 2023 [47]	Karaganda	Case–control	Diabetic nephropathy	109	n/a	0–17 years	n/a	n/a	75 (69)
Rysbekov, 2024 [48]	Astana	Case–control	Healthy (a) *H. pylori* present (b)	65 (a) 63 (b)	29 (a) 20 (b)	10–14 years (a) 11–15 years (b)	n/a	n/a	51 (78) (a) 35 (56) (b)
Zhumalina, 2024 [49]	Aktobe	Cross-sectional	Healthy	198	98	6.2 ± 2.4 months	n/a	LC-MS/MS methodology	58 (29)
Zhumalina, 2024 [50]	Aktobe	Cross-sectional	7–12 months (a) 1–6 months (b) 0–28 days (c) Healthy	32 (a) 27 (b) 45 (c)		7–12 months (a) 1–6 months (b) 0–28 days (c)	34 ± 6 (a) 21 ± 2 (b) 13 ± 5 (c)	ECLIA	19 (59) (a) 23 (85) (b) 39 (87) (c)

Abbreviations: 25(OH)D—25-hydroxyvitamin D; CKD—chronic kidney disease; ECLIA—electrochemiluminescent immunoassay; H—healthy; *H. pylori*—*Helicobacter pylori*; LC-MS/MS—Liquid Chromatography–Tandem Mass Spectrometry; n/a—not available; RRI—recurrent respiratory infection; SD—standard deviation; VDD—vitamin D deficient.

**Table 2:** Newcastle–Ottawa Scale Results for Risk of Bias Assessment.

First Author, Year	Selection	Comparability	Exposure	Total
Study design: Case–control				
Zhumalina, 2019 [40]	3	1	3	7
Dyussenova, 2023 [45]	3	1	3	7
Taiorazova, 2023 [46]	4	1	3	8
Zeinebekova, 2023 [47]	4	1	3	8
Rysbekov, 2024 [48]	3	1	3	7
Study design: Cross-sectional				
Balmukhanova, 2021 [41]	3	1	2	6
Dyussenova, 2021 [42]	3	1	2	6
Zhamankulov, 2021 [43]	2	1	2	5
Nakipov, 2022 [44]	2	1	2	5
Zhumalina, 2024 [49]	3	1	2	6
Zhumalina, 2024 [50]	2	1	2	5

## Data Availability

The original contributions presented in this study are included in the article. Further inquiries can be directed to the corresponding author.

## References

[1] Roth D.E., Abrams S.A., Aloia J., Bergeron G., Bourassa M.W., Brown K.H., Calvo M.S., Cashman K.D., Combs G., De-Regil L.M. (2018). Global Prevalence and Disease Burden of Vitamin D Deficiency: A Roadmap for Action in Low- and Middle-Income Countries. Ann. N. Y. Acad. Sci..

[2] Bouillon R., Marcocci C., Carmeliet G., Bikle D., White J.H., Dawson-Hughes B., Lips P., Munns C.F., Lazaretti-Castro M., Giustina A. (2019). Skeletal and Extraskeletal Actions of Vitamin D: Current Evidence and Outstanding Questions. Endocr. Rev..

[3] Minich D.M., Henning M., Darley C., Fahoum M., Schuler C.B., Frame J. (2022). Is Melatonin the “Next Vitamin D”?: A Review of Emerging Science, Clinical Uses, Safety, and Dietary Supplements. Nutrients.

[4] van der Pligt P., Willcox J., Szymlek-Gay E.A., Murray E., Worsley A., Daly R.M. (2018). Associations of Maternal Vitamin D Deficiency with Pregnancy and Neonatal Complications in Developing Countries: A Systematic Review. Nutrients.

[5] Ni M., Zhang Q., Zhao J., Shen Q., Yao D., Wang T., Liu Z. (2021). Relationship between Maternal Vitamin D Status in the First Trimester of Pregnancy and Maternal and Neonatal Outcomes: A Retrospective Single Center Study. BMC Pediatr..

[6] Stoica A.B., Mărginean C. (2023). The Impact of Vitamin D Deficiency on Infants’ Health. Nutrients.

[7] Mailhot G., White J.H. (2020). Vitamin D and Immunity in Infants and Children. Nutrients.

[8] Ma S., Li W., Tojibaev K.S., Turginov O., Yang W., Ma K. (2024). Regionwide and Nationwide Floristic Richness Reveal Vascular Plant Diversity in Central Asia. Plants.

[9] Gromova O., Doschanova A., Lokshin V., Tuletova A., Grebennikova G., Daniyarova L., Kaishibayeva G., Nurpeissov T., Khan V., Semenova Y. (2020). Vitamin D Deficiency in Kazakhstan: Cross-Sectional Study. J. Steroid Biochem. Mol. Biol..

[10] Yerezhepov D., Gabdulkayum A., Akhmetova A., Kozhamkulov U.A., Rakhimova S.E., Kairov U.Y., Zhunussova G., Kalendar R.N., Akilzhanova A. (2024). Vitamin D Status, VDR, and TLR Polymorphisms and Pulmonary Tuberculosis Epidemiology in Kazakhstan. Nutrients.

[11] Akhmetova V., Balji Y., Kandalina Y., Iskineyeva A., Mukhamejanova A., Baspakova A., Uzakov Y., Issayeva K., Zamaratskaia G. (2024). Self-Reported Consumption Frequency of Meat and Fish Products among Young Adults in Kazakhstan. Nutr. Health.

[12] Rebezov M., Nikitin Y., Temerbayeva M., Uryumtseva T. (2020). Current State and Prospects of Fortified Food Production in Russia and Kazakhstan. Bull. Innov. Univ. Eurasia.

[13] Karimova G.Z., Khaimah A., Aidana Rassilbay U., Sauers D.A. (2017). Lingerie and Morality: Generation Y Kazakhstani Women’s Attitude Toward Lingerie. J. East. Eur. Cent. Asian Res..

[14] Karibayeva I., Bilibayeva G., Yerzhanova A., Alekesheva R., Iglikova A., Maxudova M., Ussebayeva N. (2024). Prevalence of Vitamin D Deficiency Among Adults in Kazakhstan: A Systematic Review and Meta-Analysis. Medicina.

[15] Клинические Прoтoкoлы МЗ РК Рахит. https://diseases.medelement.com/disease/%D1%80%D0%B0%D1%85%D0%B8%D1%82/14337.

[16] Jazayeri M., Moradi Y., Rasti A., Nakhjavani M., Kamali M., Baradaran H.R. (2018). Prevalence of Vitamin D Deficiency in Healthy Iranian Children: A Systematic Review and Meta-Analysis. Med. J. Islam. Repub. Iran.

[17] Mogire R.M., Mutua A., Kimita W., Kamau A., Bejon P., Pettifor J.M., Adeyemo A., Williams T.N., Atkinson S.H. (2020). Prevalence of Vitamin D Deficiency in Africa: A Systematic Review and Meta-Analysis. Lancet Glob. Health.

[18] Alpdemir M., Analysis M., Fatih Alpdemir M. (2019). Vitamin D Deficiency Status in Turkey: A Meta-Analysis. Int. J. Med. Biochem..

[19] Karibayeva I., Abydynova A. Serum Vitamin D Level Among Adults, Adolescents, and Children in Kazakhstan: A Systematic Review and Meta-Analysis of Published Studies: CRD42024598871. https://www.crd.york.ac.uk/prospero/#recordDetails.

[20] Karibayeva I., Bilibayeva G. Prevalence of Vitamin D Deficiency Among Adults in Kazakhstan: A Systematic Review and Meta-Analysis: CRD42024610447. https://www.crd.york.ac.uk/prospero/#recordDetails.

[21] Amir-Behghadami M., Janati A. (2020). Population, Intervention, Comparison, Outcomes and Study (PICOS) Design as a Framework to Formulate Eligibility Criteria in Systematic Reviews. Emerg. Med. J..

[22] Page M.J., McKenzie J.E., Bossuyt P.M., Boutron I., Hoffmann T.C., Mulrow C.D., Shamseer L., Tetzlaff J.M., Akl E.A., Brennan S.E. (2021). The PRISMA 2020 Statement: An Updated Guideline for Reporting Systematic Reviews. BMJ.

[23] Wells G., Shea B., O’Connell D., Peterson J., Welch V., Losos M., Tugwell P. Ottawa Hospital Research Institute. https://www.ohri.ca/programs/clinical_epidemiology/oxford.asp.

[24] Posit Team RStudio: Integrated Development Environment for R. http://www.posit.co/.

[25] Harrer M., Cuijpers P., Furukawa T.A., Ebert D.D. (2021). Doing Meta-Analysis with R: A Hands-On Guide.

[26] Amanzholkyzy A., Nurgaliyeva R.E., Kaldybayeva A.T., Batyrova T.Z., Balmaganbetova F.K., Aibassova Z.A. (2019). Biochemical Variability of Vitamin D Receptor (Vdr) Gene and Its Relationship with Bone Mineral Density in Children of the Western Region of the Republic of Kazakhstan. Res. J. Pharm. Technol..

[27] Amanzholkyzy A., Donayeva A., Kulzhanova D., Abdelazim I.A., Abilov T., Baubekov Z., Samaha I.I. (2023). Relation between Vitamin D and Adolescents’ Serum Prolactin. Prz. Menopauzalny.

[28] Donayeva A., Amanzholkyzy A., Abdelazim I.A., Rakhyzhanova S., Mannapova A., Abilov T., Khamidullina Z., Bimagambetova K., Gubasheva G., Kulzhanova D. (2024). The Relationship between Vitamin D and Adolescents’ Parathyroid Hormone and Bone Mineral Density. Prz. Menopauzalny.

[29] Donayeva A., Amanzholkyzy A., Abdelazim I., Kurmangazin M., Khamidullina Z., Kurmanalina M., Sumanova A., Shabanbayeva Z., Baubekov Z., Bissaliyev B. (2023). The Effect of Vitamin D on Adolescents’ Primary Dysmenorrhea. J. Med. Life.

[30] Donayeva A., Kulzhanova D., Amanzholkyzy A., Abdelazim I.A., Abilov T., Baubekov Z., Samaha I.I. (2023). Relationship between Vitamin D and Adolescents’ Hypothyroidism—A Cross-Sectional Study. Prz. Menopauzalny.

[31] Donayeva A., Amanzholkyzy A., Abdelazim I.A., Saparbayev S., Nurgaliyeva R., Kaldybayeva A., Zhexenova A., Stankevicius E., Khamidullina Z., Gubasheva G. (2023). The Relation between Vitamin D and the Adolescents’ Mid-Luteal Estradiol and Progesterone. Eur. Rev. Med. Pharmacol. Sci..

[32] Zhumalina A.K., Kim I.S., Delyagin W.M. (2023). Vitamin D Level and Indicators of Bone Tissue Metabolism in Kazakh Infants. Russ. Fam. Dr..

[33] Myrzabekova G.T., Rabandiyarov M.R., Suleimanova S.B., Zhubanysheva K.B., Kalakova A.A. (2021). Assessment of Vitamin D Status and Respiratory Disease Risk Factors in Children. Interdiscip. Approaches Med..

[34] Gordiyenko M., Dyussenova S.B., Kunts E.A., Sarmankulova G.A., Kurilova V. (2020). Vitamin D Deficiency in Children with Chronic Renal Disease. Med. Ecol..

[35] Taiorazova G., Alimbaeva A., Tanatarov S., Smailova Z., Lobanov Y., Ailbayeva N.M., Berikuly D., Akhmetzhanova D., Imanbayeva D. (2022). Leading Antenatal Factors of Congenital Pneumonia in Premature Newborns with Vitamin D Deficiency. Sci. Healthc..

[36] Dyussenova S., Isayev V., Bukayev E. (2022). Analysis of the Relationship between Vitamin D and CKD. Med. Ecol..

[37] Zhamankulov A., Rozenson R., Morenko M., Meral G., Akhmetova U. (2020). Recurrent Respiratoryinfections in Children. Astana Med. J..

[38] Amanzholkyzy A., Nurgalieva R.E., Dosimov A.Z., Stankevicius E., Kaldybaeva A.T. (2018). Ethnic Manifestations of Gene Polymorphisms of Vitamin D Receptor (VDR) in Adolescents of Western Kazakhstan Region. J. Natl. Med. Assoc..

[39] Hearst M.O., Himes J.H., Johnson D.E., Kroupina M., Syzdykova A., Aidjanov M., Sharmonov T. (2014). Growth, Nutritional, and Developmental Status of Young Children Living in Orphanages in Kazakhstan. Infant Ment. Health J..

[40] Zhumalina A.K., Bekmukhambetov E.Z., Tusupkaliev B.T., Zharlikasinova M.B. (2019). Development of Scientifically Justified Proposals on the Prevention and Treatment of Environmentally Determined Constitutional Growth Delay in Children in the West Kazakhstan Region. Environ. Geochem. Health.

[41] Balmukhanova A., Kabulbayev K., Alpay H., Kanatbayeva A., Balmukhanova A. (2020). FGF-23 and Phosphate in Children with Chronic Kidney Disease: A Cross-Sectional Study in Kazakhstan. Medicina.

[42] Dyussenova S.B., Gordiyenko M.Y., Serikova G.B., Turlybekova S.A., Issayeva A.A., Yerimbetova N.A., Goroshko V.O. (2021). Vitamin D Deficiency in Children with Chronic Renal Disease. Open Access Maced. J. Med. Sci..

[43] Zhamankulov A., Rozenson R., Morenko M., Shnayder K., Akhmetova U., Tyo A. (2021). COVID-19 and Recurrent Respiratory Infections in Children of Kazakhstan. Russ. Open Med. J..

[44] Nakipov Z., Tursynbekova A., Dauletova G., Mussakhanova A., Dossybayeva G., Kerimbayeva Z., Saurbayeva G., Kaliyeva A., Turgambayeva A., Yen M. (2022). A Pilot Study of Nutrition Management in the Department of Pediatric Oncology Department of a Hospital in Kazakhstan. Open Access Maced. J. Med. Sci..

[45] Dyussenova S.B., Sarmankulova G.A., Sabiyeva M.M., Tlegenova K.S., Kurilova V. (2023). V The Role of Vitamine d in the Clinic of Chronic Kidney Disease in Children. Sci. Healthc..

[46] Taiorazova G., Alimbayeva A., Tanatarov S. (2023). The Role of Vitamin D and Trace Elements in Premature Newborns with Congenital Pneumonia. Bratisl. Med. J..

[47] Zeinebekova A.B., Umarova A.M., Usmanova D.U., Turkara A.M., Kovalchuk V.E., Dyussenova S.B. (2023). Early Predictors of Kidney Damage in Children and Adolescents with Type 1 Diabetes Mellitus. Clin. Nephrol..

[48] Rysbekov K., Abdrakhmanova S., Satybaeva R., Babenko D., Abdikadyr Z. (2024). Connection of Vitamin D Levels in Blood Serum with Helicobacter Pylori Infection in Paediatric Patients. Gastroenterol. Rev./Przegląd Gastroenterol..

[49] Zhumalina A., Tusupkaliev B., Mania A., Kim I., Zharlykasinova M. (2024). The Importance of Determining the Level of Bone Metabolism Markers and Vitamin D in the First Year of Life in the Kazakh Population. J. Pediatr. Pharmacol. Ther..

[50] Zhumalina A., Kim I., Tusupkaliev B., Zharlykasinova M., Zhekeyeva B. (2024). Features of D-Vitamin Status in Young Children in the Kazakh Population. Pol. Merkur. Lek..

[51] Resolution of the Government of the Republic of Kazakhstan No. 945 On the Approval of the Concept for the Development of Healthcare in the Republic of Kazakhstan Until 2026. https://adilet.zan.kz/rus/docs/P2200000945.

[52] Durá-Travé T., Gallinas-Victoriano F. (2024). Dental Caries in Children and Vitamin D Deficiency: A Narrative Review. Eur. J. Pediatr..

[53] Xiao P., Cheng H., Wang L., Hou D., Li H., Zhao X., Xie X., Mi J. (2023). Relationships for Vitamin D with Childhood Height Growth Velocity and Low Bone Mineral Density Risk. Front. Nutr..

[54] Zisi D., Challa A., Makis A. (2019). The Association between Vitamin D Status and Infectious Diseases of the Respiratory System in Infancy and Childhood. Hormones.

[55] Melough M.M., Murphy L.E., Graff J.C., Derefinko K.J., Lewinn K.Z., Bush N.R., Enquobahrie D.A., Loftus C.T., Kocak M., Sathyanarayana S. (2021). Maternal Plasma 25-Hydroxyvitamin D during Gestation Is Positively Associated with Neurocognitive Development in Offspring at Age 4–6 Years. J. Nutr..

[56] Raulio S., Erlund I., Männistö S., Sarlio-Lähteenkorva S., Sundvall J., Tapanainen H., Vartiainen E., Virtanen S.M. (2017). Successful Nutrition Policy: Improvement of Vitamin D Intake and Status in Finnish Adults over the Last Decade. Eur. J. Public Health.

[57] Płudowski P., Kos-Kudła B., Walczak M., Fal A., Zozulińska-Ziółkiewicz D., Sieroszewski P., Peregud-Pogorzelski J., Lauterbach R., Targowski T., Lewiński A. (2023). Guidelines for Preventing and Treating Vitamin D Deficiency: A 2023 Update in Poland. Nutrients.

[58] Office of Dietary Supplements Vitamin D Initiative. https://ods.od.nih.gov/Research/VitaminD.aspx.

[59] Brown L.L., Cohen B., Tabor D., Zappalà G., Maruvada P., Coates P.M. (2018). The Vitamin D Paradox in Black Americans: A Systems-Based Approach to Investigating Clinical Practice, Research, and Public Health—Expert Panel Meeting Report. BMC Proc..

[60] Saberi-Karimian M., Ghazizadeh H., Zanganeh Baygi M., Minaie M., Sadeghi F., Pouraram H., Elmadfa I., Esmaily H., Khadem Rezaian M., Tavallaei S. (2023). The National Health Program for Vitamin D Supplementation in a Developing Country. Clin. Nutr. ESPEN.

[61] Aghapour B., Kheirouri S., Alizadeh M., Khodayari-Zarnaq R. (2023). Vitamin D Deficiency Prevention Policies in Iran: A Retrospective Policy Analysis. Front. Nutr..

[62] Rostami M., Tehrani F.R., Simbar M., Yarandi R.B., Minooee S., Hollis B.W., Hosseinpanah F. (2018). Effectiveness of Prenatal Vitamin D Deficiency Screening and Treatment Program: A Stratified Randomized Field Trial. J. Clin. Endocrinol. Metab..

[63] Canadian Paediatric Society Preventing Symptomatic Vitamin D Deficiency and Rickets Among Indigenous Infants and Children in Canada. https://cps.ca/documents/position/vitamin-d-deficiency-and-rickets-among-indigenous-infants-and-children.

[64] Slater J., Larcombe L., Green C., Slivinski C., Singer M., Denechezhe L., Whaley C., Nickerson P., Orr P. (2013). Dietary Intake of Vitamin D in a Northern Canadian Dené First Nation Community. Int. J. Circumpolar Health.

[65] Altieri B., Cavalier E., Bhattoa H.P., Perez-Lopez F.R., Lopez-Baena M.T., Perez-Roncero G.R., Chedraui P., Annweiler C., Della Casa S., Zelzer S. (2020). Vitamin D Testing: Advantages and Limits of the Current Assays. Eur. J. Clin. Nutr..

[66] Fraser W.D., Milan A.M. (2013). Vitamin D Assays: Past and Present Debates, Difficulties, and Developments. Calcif. Tissue Int..

